# Improved Focalization of Electrical Microstimulation Using Microelectrode Arrays: A Modeling Study

**DOI:** 10.1371/journal.pone.0004828

**Published:** 2009-03-12

**Authors:** Sébastien Joucla, Blaise Yvert

**Affiliations:** Université de Bordeaux, CNRS, Centre de Neurosciences Intégratives et Cognitives, UMR Talence, France; Vrije Universiteit Amsterdam, Netherlands

## Abstract

Extracellular electrical stimulation (EES) of the central nervous system (CNS) has been used empirically for decades, with both fundamental and clinical goals. Currently, microelectrode arrays (MEAs) offer new possibilities for CNS microstimulation. However, although focal CNS activation is of critical importance to achieve efficient stimulation strategies, the precise spatial extent of EES remains poorly understood. The aim of the present work is twofold. First, we validate a finite element model to compute accurately the electrical potential field generated throughout the extracellular medium by an EES delivered with MEAs. This model uses Robin boundary conditions that take into account the surface conductance of electrode/medium interfaces. Using this model, we determine how the potential field is influenced by the stimulation and ground electrode impedances, and by the electrical conductivity of the neural tissue. We confirm that current-controlled stimulations should be preferred to voltage-controlled stimulations in order to control the amplitude of the potential field. Second, we evaluate the focality of the potential field and threshold-distance curves for different electrode configurations. We propose a new configuration to improve the focality, using a ground surface surrounding all the electrodes of the array. We show that the lower the impedance of this surface, the more focal the stimulation. In conclusion, this study proposes new boundary conditions for the design of precise computational models of extracellular stimulation, and a new electrode configuration that can be easily incorporated into future MEA devices, either *in vitro* or *in vivo*, for a better spatial control of CNS microstimulation.

## Introduction

Electrical extracellular stimulation of the central nervous system has been used empirically for several decades by electrophysiologists to explore fundamental properties of neural networks. Currently, peripheral nerve, deep brain, and spinal cord stimulation paradigms are also used routinely for clinical restoration of lost motor function [1] and treatments of neurological disorders such as neuropathic pain [2], movement disorders, Parkinson disease [3], or epilepsy [4]. These healing strategies mainly use macroscopic implanted electrodes of several mm^2^ to stimulate large regions of the central nervous system. More recently, microstimulation, which makes use of electrodes on the µm scale, is gaining increasing interest in both fundamental and clinical research, opening the possibility to stimulate small groups of neurons instead of large regions. In this perspective, microelectrode arrays (MEAs) are the focus of intensive developments [5]–[8]. These *in vitro* or *in vivo* microsystems increasingly benefit fundamental neuroscience aiming at understanding activity-dependent plasticity of neural networks, as well as clinical developments of efficient neural implants or prostheses [9], [10].

As reported recently, the activation of single neurons may strongly impact the activity of a large neural network and even behavior [11], [12]. Such data illustrate the fact that future developments of efficient MEA stimulation devices will require precise activation of small groups of neurons, so that each electrode of an array will act as an independent “stimulation pixel”, directly influencing cells in its close vicinity, and not those located in the vicinity of other electrodes. For this reason, determining optimal electrode configurations for efficient stimulation is still the focus of current developments based on modeling approaches [13]–[16], where compartimentalized neurons are stimulated by modeled extracellular potential fields. The potential field is calculated by solving the homogeneous Poisson equation under appropriate boundary conditions. Solutions to this problem can be derived analytically when the volume geometry and electrode configuration are simple [17]–[21]. However, when realistic geometries are considered, numerical simulations are required, such as finite element or finite difference models [14], [22]–[24].

The aim of the present study is twofold: First, we validate a finite element model (FEM) for the realistic computation of the electrical potential field, and, second, we propose a new electrode configuration to achieve focal stimulations of neural networks using MEAs. This paper is thus divided into two parts. In the first part, we developed a FEM for the calculation of the potential field incorporating the surface conductance of the electrodes through Robin boundary conditions, which we validated on experimental recordings of the electrical potential field. In the second part of the paper, we used this model to evaluate the focality of MEA stimulations for different electrode configurations, in terms of both the potential field and the threshold-distance curves for a straight fiber and a reconstructed cortical neuron. In particular, we propose a variant of the monopolar configuration consisting in replacing the usual distant ground electrode by a ground surface surrounding all the electrodes of the array. We show that this new configuration improves the stimulation focality, and that this improvement is best when the interface conductance of this ground surface is high. This configuration can easily be incorporated into microelectrode arrays for *in vitro* applications, as well as *in vivo* neuroprosthetic devices requiring focal stimulations. Part of this work has been presented in abstract form [25] and a patent application has been submitted for the new electrode configuration.

## Methods

### 1. Experimental MEA recordings of the extracellular potential field generated by an electrical stimulation

Using microelectrode arrays (MEAs) dedicated to *in vitro* experiments, we recorded the electrical potential distribution induced by extracellular stimulation. Current-controlled stimulations were delivered either in the absence (Ringer only) or in the presence of neural tissue. Experimental protocols conformed to recommendations of the European Community Council and NIH Guidelines for care and use of laboratory animals.

#### a. Microelectrode array

**Figure 1:**
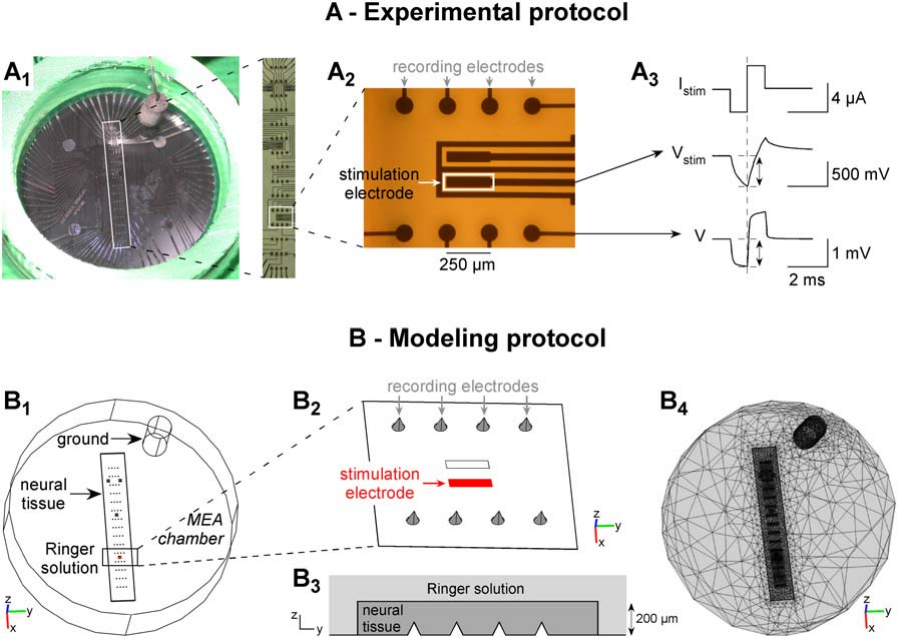
Experimental (A) and modeling (B) stimulation paradigms. A: Top view of the experimental MEA chamber (A1). The “spinal MEA” from Ayanda BioSystems consists of 4×15 3D recording electrodes (arrows in A2) and 8 2D stimulation electrodes (rectangles in A2). Current-controlled monopolar stimulations were applied between a 2D stimulation electrode and an external cylindrical Ag/AgCl ground electrode. The potential field *V* generated in the medium was measured by the 60 recording microelectrodes, and the stimulation electrode voltage (*V_metal_* = *V_stim_*) was recorded with a home-made follower circuit. Peak values were measured at the end of the cathodic phase of the pulse stimulus (see example in A3). B: Top view of the 3D finite element model used for the computation of the potential field. The 60 recording electrodes were modeled by cones, and the stimulation electrode by a 2D rectangular surface on the substrate (B2). All these electrodes were represented as surface boundaries in the finite element model. When present, the neural tissue was modeled as a 200-µm-thick parallelepiped with a different electrical conductivity than that of the Ringer solution (B3). The 3D mesh consisted of 63,214 tetrahedral Lagrange P2 elements, corresponding to 101,105 degrees of freedom (B4).

We used a microelectrode array to deliver electrical stimulations and to record the potential field induced in the MEA chamber (Figure 1.A1). The array was composed of a 4×15 grid of 3D recording microelectrodes (base diameter: 80 µm, height: 80 µm, width spacing: 250 µm, length spacing: 750 µm), 8 2D rectangular stimulation electrodes (60×250 µm^2^), and 4 integrated ground disk electrodes (diameter 1 mm), all made of Pt (*Ayanda Biosystems*, Lausanne, Switzerland). The 4 integrated ground electrodes were disconnected and not used in this study. Instead, an external cylindrical Ag/AgCl ground electrode pellet (diameter: 2 mm, height: 4.3 mm) was used (*World Precision Instruments*, Aston, England). The array was surrounded by a cylindrical glass chamber, and the bottom part, including electrode leads, was insulated from the extracellular medium by a 5 µm thick SU-8 epoxy layer [26].

#### b. Experimental preparation

Stimulations were first performed in a Ringer solution composed of (in mM): 113 NaCl, 4.5 KCl, 2 CaCl_2_2H_2_O, 1 MgCl_2_6H_2_O, 25 NaHCO_3_, 1 NaH_2_PO_4_H_2_O and 11 D-Glucose. We also performed stimulations in the presence of a whole embryonic mouse hindbrain-spinal cord preparation, which was dissected as described previously [27]. Briefly, E14.5 embryos were surgically removed from pregnant OF1 mice (*Charles River Laboratories*, L'Arbresle, France) previously killed by cervical dislocation. The whole spinal cord and medulla were dissected in the Ringer solution (pH 7.5) gassed with carbogen (95% O_2_, 5% CO_2_), meninges were removed, and the preparation was then placed in the MEA cylindrical chamber. A plastic net with small holes (70×70 µm^2^) was laid on the neural tissue, in order to achieve a tight and uniform contact with the microelectrodes. Experiments were performed at room temperature.

#### c. Stimulation protocols

Current-controlled monopolar stimulations were performed between one 2D stimulation electrode of the array (Figure 1.A2) and the external cylindrical ground electrode. Stimulation amplitudes and durations were chosen so that injected charges remained below the safe charge injection limits of the Pt electrodes (see http://www.ayanda-biosys.com/Documents/safe_charge_injection_limit.pdf). Stimuli consisted of a train of 10 cathodic-first biphasic current pulses separated by 10 sec (phase duration: 1 ms, amplitude *I_stim_* = ∓4 µA in the Ringer solution and *I_stim_* = ∓1 µA in the presence of a neural tissue to avoid saturation of the amplifiers). They were delivered using the STG2008 stimulator controlled by the MC_Stimulus II v2.1.4 software (*Multi Channel Systems*, Reutlingen, Germany).

#### d. Recordings

The electrical potential field was recorded on the 60 3D recording electrodes referenced to the Ag/AgCl ground electrode pellet, 1200× amplified and low-pass filtered at 3 kHz (*Multi Channel Systems* MEA1060 filter amplifiers). Also, the voltage of the stimulation electrode was measured with a home-made follower circuit. It should be noted that no 3-electrode montage was needed here because we recorded the metal voltage of the stimulation electrode (*V_stim_*) with respect to the metal voltage of the ground electrode (zero by convention). In particular, we did not measure the junction potential at the stimulation electrode interface, which would have required a 3-electrode montage, and only considered the variations of the interface potential around the junction potential. Data were acquired at 15625 Hz using the Micro 1401 AD converter and the Spike2 v5.14 software from *Cambridge Electronic Design* (Cambridge, England). Examples of recordings of the stimulation electrode voltage *V_stim_* and of the potential in the medium *V* at a recording electrode are shown in Figure 1.A3.

#### e. Data analysis

Time courses of the potential field recorded at each of the 60 electrodes, as well as the stimulation electrode voltage, were averaged across the 10 stimuli. Absolute peak values were measured at the end of the cathodic phase (as shown in Figure 1.A3), and the variance of the potential field was also calculated at this latency.

### 2. Theoretical background for the potential field modeling

Under quasi-static approximation, the electrical potential *V* in a medium of conductivity *σ* is the solution of the homogeneous Poisson equation:
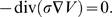
(1)The solution of this equation is defined uniquely by conditions imposed on the boundaries of the volume conductor. The quality of the solution thus depends on the pertinence of these boundary conditions (BCs) on the domain frontiers. In practice, two types of BCs can be considered, either insulating or conductive.

Insulating boundaries relate to all frontiers through which no current flows, namely:
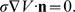
(2)This condition can thus be used for all the insulating frontiers of the medium, such as the air/medium interface and the edge and insulated floor of the chamber. We previously showed that it is also adequate for modeling the surface of recording electrodes [28].

**Figure 2:**
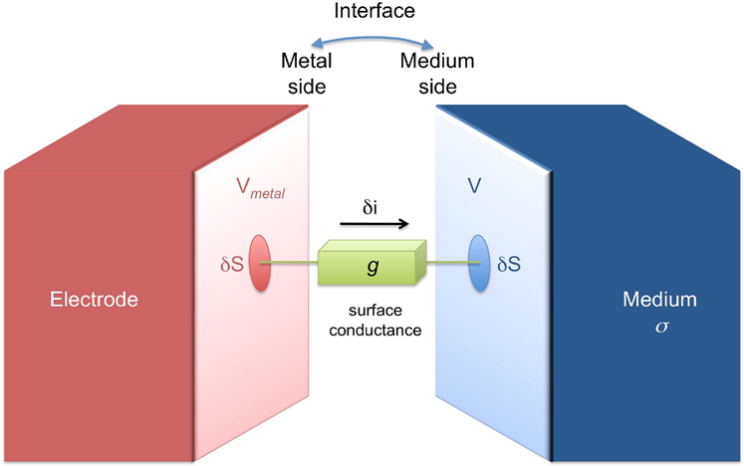
Schematic representation of the electrode/medium interface. During an electrical stimulation, a potential drop occurs between the metal and the medium sides of the interface. This drop can be modeled by a Robin boundary condition, taking into account the surface conductance of the interface (*g*) and the electrical conductivity of the medium (*σ*). While the voltage on the metal side *V_metal_* is uniform, the electrical potential in the medium side (*V*) is allowed to vary over the electrode surface. Please, note here that even in the absence of stimulation, a junction potential exists at the interface. However, in this paper, this electrochemical equilibrium potential is assumed to be constant during the short time of the stimulation, and only the variations of the potential difference at the interface during the stimulation are considered.

When modeling stimulations, a conductive boundary condition should be used on the surfaces of the electrodes through which a current flows, namely the stimulation and ground electrodes. The type of BC used on these electrodes directly determines the calculation of the potential field. To obtain an accurate calculation, it is thus crucial to choose BCs that best reflect the electrode/medium interface. Figure 2 illustrates this interface with the surface conductance *g* between the metal side and the medium side. It is important to note that, when a stimulation is imposed on an electrode, only the potential of the metal side of the electrode, *V_metal_*, can be known, while the potential *V* in the medium in front of the electrode is unknown (and is the purpose of the computation). The relationship between *V_metal_* and *V* is given by writing Ohm's law at the interface. Considering an elementary piece of surface δ*S*, the elementary current δ*i* flowing through δ*S* is given by:
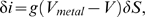
(3)where *g* is the surface conductance of the interface. Moreover, on the medium side, the current entering the medium through δ*S* is given by:
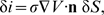
(4)where **n** is the unit vector normal to the surface. From Equations 3 and 4, the natural BC that can be applied to the frontier of the medium in front of the electrode is thus the following Robin BC:
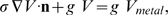
(5)This general BC has been mentioned previously [26], but not further investigated. It should be noted that the case of insulating boundaries (Equation 2) can be deduced from this relation by setting *g* = 0. In the opposite case of an infinitely conductive interface (*g*→∞), this condition reduces to the classically used Dirichlet condition:
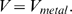
(6)However, in practice, electrodes are not infinitely conductive and have a non-zero impedance creating a potential drop at the interface, which is neglected with the Dirichlet BC. We thus built a FEM with Robin BCs (Equation 5) to take this drop into account. Also, Robin BCs do not impose the potential *V* on the medium side to be uniform in front of the electrode as with Dirichlet BCs. Indeed, since electrodes are made of metals (usually gold, platinum, or iridium) the electrode voltage on the metal side, *V_metal_*, has to be uniform. However the less conductive medium does not impose the potential *V* on the medium side to be uniform as well. We will see below that the new electrode configuration proposed in this paper actually takes advantage of this important property.

It should be noted that when a metal electrode is bathed in a conductive solution, a junction equilibrium potential establishes between both sides of the interface [29]. When a current is injected, this potential difference at the interface varies transiently according to the interface conductance (Equation 3). In the present study, we only consider the variations of the interface potential around the equilibrium. This is justified by the fact that the equilibrium potential of a metal/medium interface varies slowly (on the order of a second) compared to the transient change of the interface potential during a stimulation pulse (on the order of a ms).

### 3. Finite element modeling of the extracellular potential field generated by an electrical stimulation

We developed a 3D finite element model (FEM) in order to compute the electrical potential field generated in a conductive medium (Ringer only or Ringer and neural tissue) by an electrical stimulation. This model was tested to reproduce the potential field obtained experimentally. Simulations were run with the finite element simulation software FEMLAB® 3.1a (COMSOL AB, Stockholm, Sweden) interfaced with Matlab 6.2 (The Mathworks, Natick, USA), under Linux (Fedora 7).

#### a. Model geometry

The 3D model geometry corresponded to the experimental MEA, including the chamber, the neural tissue, the recording and stimulation microelectrodes of the array, and the external ground electrode pellet (see Figure 1.B1). The outer limits of the model corresponded to the inner geometry of the MEA cylindrical chamber (diameter: 19 mm, height: 5 mm). This volume was subdivided into two regions, representing the Ringer solution and the neural tissue. The neural tissue was modeled as a rectangular slab with dimensions close to that of the embryonic mouse hindbrain-spinal cord preparation (length: 13 mm, width: 2 mm, height: 200 µm, see Figure 1.B3). We verified that modeling a more realistic shape did not alter the distribution of the electrical potential within the tissue. The electrodes of the array were modeled on the bottom surface of the chamber: The 3D recording electrodes were represented by 3D conical boundaries (base diameter: 80 µm, height: 80 µm, see Figure 1.B3), and the stimulation electrodes were represented by surface rectangles (width: 60 µm, length: 250 µm). These dimensions corresponded to that of the actual MEA used experimentally (compare Figure 1.A2 and Figure 1.B2). Finally, the external ground electrode was modeled by a cavity inside the domain representing its interface with the Ringer solution (diameter: 2 mm, height: 4.3 mm).

#### b. Volume equation and boundary conditions

The finite element model solved the homogeneous Poisson equation (Equation 1). The electrical conductivities of the Ringer solution and neural tissue were supposed homogeneous and isotropic in each region. When no tissue was considered, the conductivity of the tissue region was set to that of the Ringer solution, which was measured with a conductimeter and found to be *σ_Ringer_* = 1.65 S/m at about 700 Hz and room temperature. Possible variations of conductivity with respect to frequency were neglected. When tissue was present, its conductivity was one of the parameters that was estimated to fit the experimental recordings of the electrical potential field.

Insulating BCs (Equation 2) were assigned to the circumference of the chamber, the air-Ringer solution interface (top part of the chamber), the insulated floor of the chamber, the 7 unused (and disconnected) 2D stimulation electrodes, and also the 60 3D recording electrodes. Indeed, although current may enter and exit recording electrodes at different places on their surface, on average the global current flowing through these electrodes was negligibly small due to the very high amplifier input impedance (10^13^ Ω). It should be noted that this type of BC allows the potential in front of the recording electrodes to be non-uniform.

Robin BCs (Equation 5) were used for the conductive elements (ground and stimulation electrodes). The metal voltage in Equation 5 was set to *V_metal_* = 0 for the ground and *V_metal_* = *V_stim_* = 754.4 mV (measured) for the stimulation electrode. The surface conductances of these electrodes (*g_ground_* and *g_stim_*) were optimized so that the modeled potential field best fitted the experimental one.

#### c. Mesh and solver

The 3D geometry of the model was meshed with 63,214 tetrahedral Lagrange P2 elements, corresponding to 101,105 degrees of freedom (Figure 1.B4). The problem was solved by direct inversion of the finite element matrix (mode *Direct (UMFPACK)*). Using this mesh, one calculation of the extracellular potential field took about 21 seconds on a Pentium IV 2.4 GHz with 2 Gb RAM. We verified that, with a finer mesh (297,156 elements, 428,290 degrees of freedom), and the SSOR-preconditioned conjugated gradient algorithm solver, the potential on the recording electrodes differed by less than 0.1%.

#### d. Integration of the electrical potential field over each recording electrode

The FEM was validated by comparing the experimental and the modeled data across all recording electrodes. Once the potential *V* in the medium has been calculated, the metal voltage *V_metal,j_* of each recording electrode *j* has to be calculated. Using Robin BCs on recording electrodes, these values would be directly estimated under the constraints that no global current flows through recording electrodes: 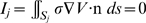. It can be noted that integrating Equation 5 under these constraints leads to:
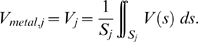
(7)However, we did not use Robin BCs on recording electrodes because this would have required to optimize simultaneously the *V_metal_* value of all electrodes (60 more parameters). Instead, we used homogeneous Neumann BCs (Equation 2), and calculated a posteriori the metal voltage of each recording electrode using Equation 7. We checked, on a single recording electrode model, that using this approach led to errors in the estimation of *V_metal_* of less that 0.1% compared to that obtained directly using a Robin BC on the recording electrode.

#### e. Estimation of the model parameters

The FEM solution depended on the following parameters: the conductivities of the Ringer solution (*σ_Ringer_*) and the neural tissue (*σ_tissue_*), and the surface conductances of the stimulation (*g_stim_*) and ground (*g_ground_*) electrodes. For all simulations, the conductivity of the Ringer solution was set to the measured value *σ_Ringer_* = 1.65 S/m. The other parameters were optimized to best fit experimental recordings of the potential field, using the Levenberg-Marquardt algorithm to minimize the following weighted least squares criterion:
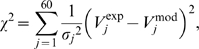
(8)where *σ_j_* ^2^ was the measured variance of the experimental potential *V_j_* ^exp^.

#### f. Determination of the model quality

The model quality was determined by performing a linear regression between the modeled and experimental potential fields. A perfect modeling would lead to a linear regression with a slope of 1 and an intercept of 0.

### 4. Comparison of the stimulation focality for different electrode configurations

We further used a FEM to predict and compare the focality of the electrical potential field and threshold-distance curves for different electrode configurations.

#### a. Electrode configurations and model geometry

**Figure 3:**
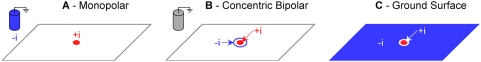
Electrode configurations considered for the stimulation focality evaluation. The focality of the potential field and threshold-distance curves were determined for three electrode configurations: Monopolar (M), concentric bipolar (CB), and a new configuration in which the external ground was replaced by a ground surface (GS) integrated on the substrate of the array and surrounding the microelectrodes. The different configurations are simply selected by assigning adequate boundary conditions to the different electrodes: Robin BCs (Equation 5) for conductive electrodes, homogeneous Neumann BCs (Equation 2) for insulating boundaries.

We tested three electrode configurations (Figure 3): monopolar, concentric bipolar, and a new configuration where the distant ground electrode is replaced by a ground surface integrated on the array and surrounding the microelectrodes. For these simulations, we considered a planar MEA, which is of the type of the electrodes used for *in vitro* chronic stimulation of neural networks maintained in culture. Simulations were thus performed using a different model geometry than the one described above. For monopolar stimulations, a planar disk electrode (diameter 10 µm, Figure 3.A) and a 3D cylindrical ground electrode, similar to the one used in the previous geometry, were used. For concentric bipolar, one annular electrode (inner diameter: 25 µm, radial width: 3 µm, as used by Edell et al. [30]) was added around the disk electrode to ensure the complete return of the current (Figure 3.B). The external ground electrode was kept in this case although no current returned through this electrode. For the new configuration, the cylindrical ground was replaced by a ground surface with a 25-µm-diameter opening surrounding the disk electrode (Figure 3.C). The corresponding mesh consisted of 959,122 tetrahedra and 1,323,271 degrees of freedom. The problem was solved using an SSOR-preconditioned conjugated gradient algorithm.

#### b. Model parameters and boundary conditions

The volume conductivity was uniformly set to 1.95 S/m, corresponding to 37°C, the practical temperature for *in vitro* cultures or *in vivo* experiments. All electrodes were equipped with Robin BCs with parameter values corresponding to the ones previously fitted to reproduce experiment potential field. The stimulating electrodes had a surface conductance *g_stim_* = 338 S/m^2^, and a metal voltage value *V_stim_* corresponding to a current of 1 µA. The cylindrical ground electrode had a surface conductance *g_ground_* = 975 S/m^2^ and a metal voltage of 0 V. For the CB configuration, the metal voltage of the annular counter-electrode was chosen to ensure complete return of the stimulation current. Finally, when a ground surface (GS) was considered, no other external ground electrode was used. The metal voltage of the GS was set to 0 V and several values of surface conductance were tested: *g_GS_* = 400, 4000, 40000 S/m^2^ or infinite (homogeneous Dirichlet condition *V* = 0). We also tested in Figure 8 the focality obtained using a non homogeneous Neumann BC on the ground surface (see Equation 13 and comments on this point in the discussion).

#### c. Comparison of the potential field focality for the three electrode configurations

We first compared the focality of the potential field on a horizontal plane at *z* = 50 µm above the stimulating disk electrode. For this purpose, the potential field was normalized. Indeed, as we shall see below (see Figure 6.A), changing the surface conductance of the ground electrode modifies the global offset of the potential field. This is the case for the monopolar and GS configurations but not for the concentric bipolar configuration for which no current returns through the ground electrode. This offset was substracted from the potential field in order to be able to compare the different configurations. We further normalized the potential field by its maximum value over the plane at *z* = 50 µm. The resulting normalized potential field was thus a number between 0 and 1, defined as follows:
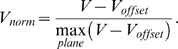
(9)


#### d. Comparison of the stimulation focality for the three electrode configurations

We also performed numerical simulations with compartmentalized neurons embedded in the extracellular potential fields to further assess the stimulation focality of the three configurations. These simulations were performed with the NEURON software, v6.1 [31]. We considered both a straight fiber (Figure 9.A) and a complex CNS neuron, taken from the literature [32] and obtained from ModelDB (accession number 2448) (Figure 9.B). The straight fiber (length: 1 mm, diameter: 1 µm) was equipped with standard Hodgkin-Huxley [33] active currents implemented by default in the NEURON environment (leakage conductance = 3×10^−4^ S/cm^2^, sodium conductance = 0.12 S/cm^2^, potassium conductance = 0.036 S/cm^2^, membrane capacitance = 1 µF/cm^2^, intracellular resistivity = 100 Ω.cm, resting potential = −70 mV), while the layer IV stellate cell model was used as is (its axon was straight with a diameter of 0.6–0.8 µm). Temperature was set to 37°C for the calculation of voltage-dependent conductances. For each neuron and each stimulation configuration, the extracellular potential computed in the finite element model (without offset correction) was interpolated at the center of each compartment and assigned with the extracellular mechanism. This approach, which has been used by others and in a previous study [34], is detailed in the NEURON documentation (available online at http://www.neuron.yale.edu/neuron/docs/help/neuron/neuron/mech.html#extracellular). Cathodic-first biphasic stimulations (phase duration: 200 µs) were used, the amplitude of which was increased until firing an action potential (detected at the middle of the fiber, or at the first node of Ranvier for the stellate cell). A 10-µs time step was used, allowing a reduced error on the activation threshold estimation (using a 10 times smaller time step led to a threshold difference of less than 1%). The stimulation focality of the three configurations was assessed by moving both structures on a horizontal line passing over the stimulating electrode (*z* = 50 µm for the straight fiber, *z* = 110 µm for the stellate cell) and determining the activation thresholds along the line.

## Results

### 1. Validation of the FEM calculation of the potential field using experimental recordings

**Figure 4:**
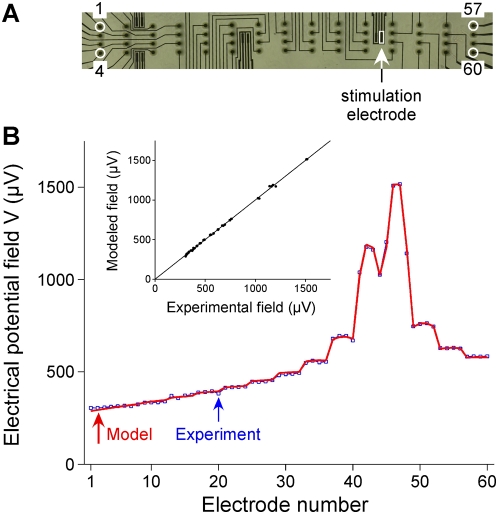
Validation of the extracellular potential field modeling using Robin boundary conditions. A: Microphotograph of the microelectrode array, with the 4×15 recording electrodes numbered from 1 to 60 (from top left to bottom right). Current-controlled monopolar stimulations were applied in a Ringer solution between the stimulation electrode (indicated by an arrow) and the external ground electrode. B: The electrical potential field recorded experimentally (square symbols) was modeled (red curve) using the finite element model (described in Figure 1.B) equipped with Robin BCs on the conductive electrodes. The surface conductances of the stimulation and ground electrodes (*g_stim_* and *g_ground_*) were estimated to optimize the fit between modeled and experimental fields. The linear regression between the modeled and the experimental fields is shown in the inset (R^2^ = 0.999, p<0.0001).

Monopolar stimulations were applied in a Ringer solution between a 2D stimulation microelectrode of the array and an external cylindrical ground electrode, and the potential field was measured on the 60 recording electrodes of the array. The FEM solved the homogeneous Poisson equation (Equation 1) under given boundary conditions to reproduce these experimental data. We first tested the use of a standard Dirichlet BC (Equation 6 with *V_metal_* = *V_medium_* = *V_stim_* = 754.4 mV on the stimulation electrode and *V_metal_* = *V_medium_* = *V_ground_* = 0 on the ground electrode) and found that the modeled potential field was two orders of magnitude higher than the experimentally recorded one (regression slope of 107.3±0.4, and intercept of −14291±280 µV). This large difference was due to the fact that the potential drops across the stimulation and ground electrode/electrolyte interfaces were not taken into account by this type of BC. To model this potential drop we used Robin BCs (Equation 5), taking into account the surface conductance of the metal/medium interface of the stimulation and ground electrodes. This BC depends on three parameters: The stimulation electrode voltage, which was set to the measured value (*V_metal_* = *V_stim_* = 754.4 mV), and the surface conductances *g_ground_* and *g_stim_* of the electrodes which were estimated with a Levenberg-Marquardt algorithm so as to best reproduce experimental data (*g_ground_* = 975 S/m^2^, *g_stim_* = 338 S/m^2^). This model gave an excellent fit of the experimental recordings of the potential field (Figure 4), as assessed by the linear regression between the modeled and the experimental fields: slope of 1.002±0.004 and intercept of −0.0005±0.003 µV (R^2^ = 0.999, p<0.0001). Moreover, we checked for a 1000-Hz sinusoidal stimulation that the fitted value of the surface conductance of the stimulation electrode led to the prediction of a theoretical electrode impedance (60.9 kΩ) close to the actually measured impedance (65 kΩ). In the following, we thus use the model equipped with Robin boundary conditions on the stimulation and ground electrodes.

### 2. Distribution of the electrical potential over the ground and stimulation electrode surfaces

**Figure 5:**
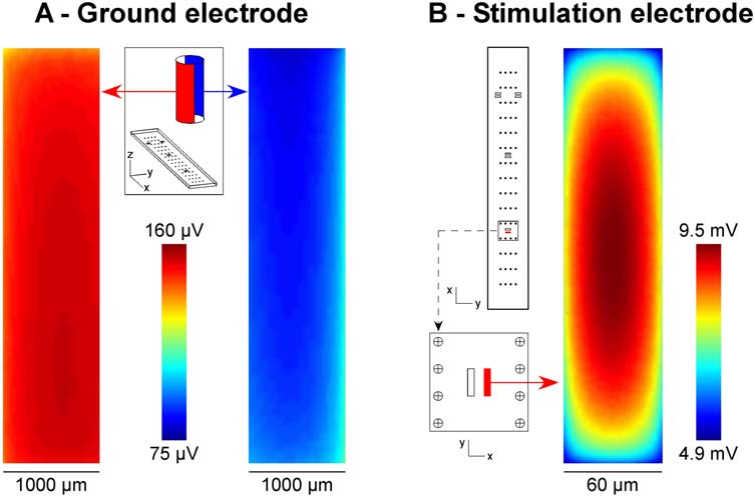
The spatial distribution of the potential in the medium is not uniform over the surfaces of the ground (A) and stimulation (B) electrodes. Over the ground electrode (A, *yz* view), *V* is two times higher on the side oriented towards the stimulation electrode (left) than on the opposite side (right). Over the stimulation electrode (B, *xy* view), *V* is two times higher at the center of the electrode than on the electrode borders.

The specific property of the Robin BC is to take into account the surface conductance of the electrode, which allows the electrical potential (in the electrolyte) to be non-uniform in front of the electrode surface. We indeed found that the potential was not uniform on both the stimulation and ground electrodes (Figure 5). Over the ground electrode surface, for which the metal voltage was 0 V, the electrolyte potential was two times higher on the side oriented towards the stimulation electrode (160 µV, Figure 5A left) than on the opposite side (75 µV, Figure 5.A right). Hence, the current flowing through the electrode, which is proportional to the potential drop across the interface, was highly non-uniform. Over the stimulation electrode surface, the electrolyte potential varied from 4.9 mV to 9.5 mV. Because these values were small compared to the metal potential of the stimulation electrode (754.4 mV), the potential drop across the interface was nearly uniform, meaning that the current flowed almost uniformly across the surface of this electrode.

### 3. Influence of the surface conductance of the electrodes on the potential field

**Figure 6:**
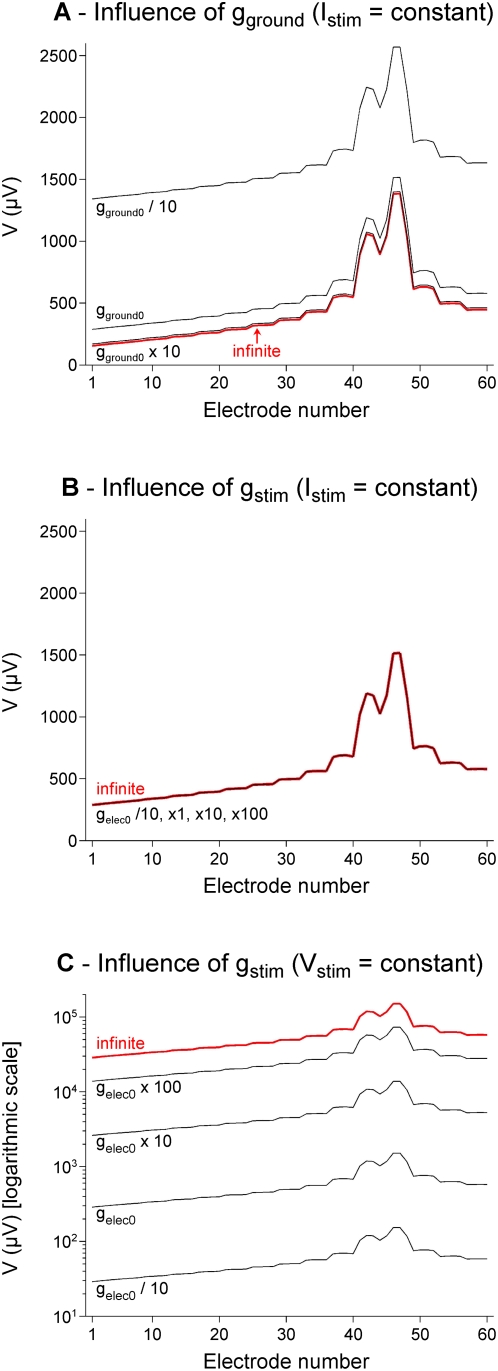
Influence of the surface conductance of the ground and stimulation electrodes on the potential field distribution. Each parameter was varied independently around its initial value fitted in Figure 4 (*g_ground_* = 795 S/m^2^, *g_stim_* = 338 S/m^2^). For current-controlled stimulations, modifying the ground electrode surface conductance *g_ground_* induces a global shift in the offset of the potential distribution. The limit case (*g_ground_*→∞) is computed using a homogeneous Dirichlet BC on the ground surface (A). For current-controlled stimulations, the potential field recorded on the 60 electrodes is insensitive to modifications of the stimulation electrode surface conductance *g_stim_* (B). By contrast, for voltage-controlled stimulations, changing *g_stim_* induces a global scaling of the potential field (C).

Figure 6 shows the influence of the ground and stimulation electrodes' surface conductance (*g_ground_* and *g_stim_*, respectively) on the spatial distribution of the potential field. Each panel shows the influence of one parameter considered separately from the other, which were set to their values fitted in Figure 4.

First, increasing the surface conductance of the ground induces a global *shift* of the potential field towards smaller values (Figure 6.A). The “limit” case, where the potential field is the lowest, is obtained for the standard homogeneous Dirichlet BC (i.e., when *g_ground_*→∞). This global offset is due to the potential drop across the ground/electrolyte interface, which decreases when *g_ground_* increases due to Ohm's law at the interface. Because the potential field is defined relative to a constant (from Equation 1), the whole field is then shifted by the value of this drop. It should be noted that *g_ground_* actually influences the shape of the potential field in the close vicinity of the ground electrode. Indeed, as *g_ground_* increases, *V* becomes all the more uniform (and close to zero) in front of the ground electrode surface. However, this influence is not seen on the recording electrodes in the case of the classical monopolar configuration. By contrast, we will see below that *g_ground_* strongly influences the shape of the potential field when the ground electrode is replaced by a ground surface surrounding the electrodes of the array.

Second, we determined the influence of the surface conductance of the stimulation electrode (*g_stim_*) for both current-controlled stimulations (where the metal voltage of the electrode is adjusted so that the current injected through the stimulation remains constant) and voltage-controlled stimulations (where the metal voltage of the electrode is fixed to *V_stim_* = 754.4 mV, and the current is not controlled). In the case of current-controlled stimulations, changing *g_stim_* has no influence on the potential field distribution (Figure 6.B). By contrast, in the case of voltage-controlled stimulation, changing *g_stim_* induces a scaling of the potential field amplitude, which increases as *g_stim_* increases (Figure 6.C), the limit case (*g_stim_*→∞) being obtained for the standard Dirichlet BC (*V_stim_* = 754.4 mV). These results mean that the field amplitude is entirely determined by the current injected through the stimulation electrode and not by its metal voltage.

It should be noted that, for current-controlled stimulations, the potential distribution in the medium is actually not uniform locally on the stimulation electrode (Figure 5.B) and this non-uniformity decreases as *g_stim_*→∞. Thus, locally in front of the stimulation electrode surface, the potential distribution does vary when *g_stim_* varies. However, these very local variations (seen up to distances on the order of a µm) are not seen on distant recording electrodes.

### 4. Influence of the presence of neural tissue

**Figure 7:**
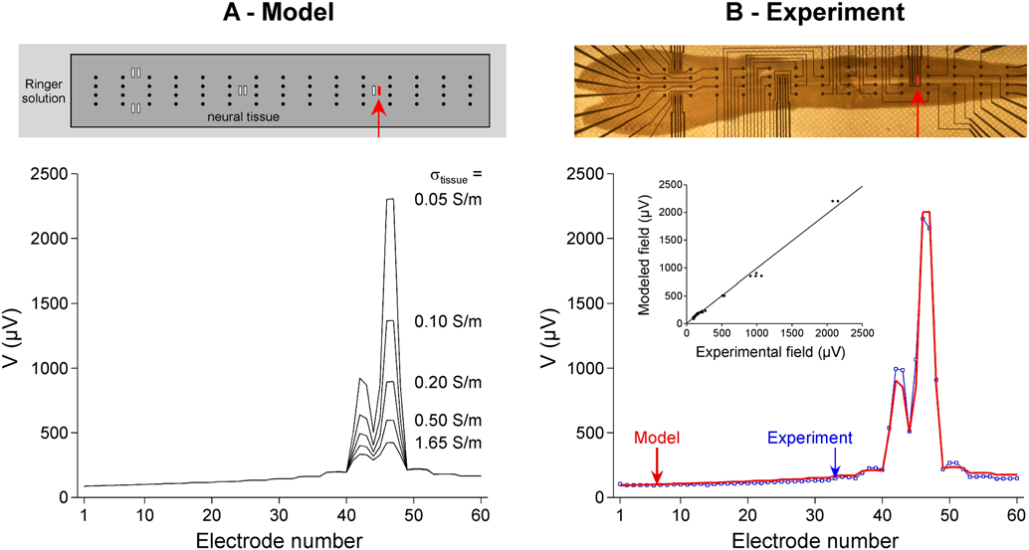
Influence of a neural tissue on the modeled (A) and experimental (B) potential fields. Current-controlled monopolar stimulations were delivered to the tissue (1 µA). A, Top: Top view of the finite element model including a neural tissue surrounded by the Ringer solution. Bottom: The potential field was computed for different values of the electrical conductivity *σ_tissue_* of the neural tissue, while the conductivity of the Ringer solution remained unchanged (*σ_Ringer_* = 1.65 S/m). B, Top: Inverted-microscope photograph of a hindbrain-spinal cord preparation on the MEA. Bottom: Experimental potential field distribution in the presence of a hindbrain-spinal cord preparation (square symbols), and modeled potential field (red curve) obtained with the finite element model equipped with Robin BCs. In this case, the surface conductances of the stimulation and ground electrodes (*g_stim_* and *g_ground_*) and the tissue conductivity *σ_tissue_* were estimated to optimize the fit between modeled and experimental fields (R^2^ = 0.989, p<0.0001). The estimated value of *σ_tissue_* was 0.057 S/m.

In the results described above, electrical stimulation was performed in the Ringer solution alone. In practice, stimulation is applied to neural tissue or cells, the presence of which changes the conductivity of the bath and thus likely influences the shape of the potential field distribution. In the case of dissociated cells, the conductivity of the bath might be close to that in the absence of cells, so that these changes should be small. However, in the case of a neural tissue (slice or whole organ), the conductivity of the extracellular space becomes smaller than that of the Ringer solution and larger changes are expected. Here we studied the influence of the presence of neural tissue on the shape of the potential field created by an electrical stimulation (Figure 7) using FEM simulations and experimental measurements.

First, we introduced a volume of neural tissue in the finite element model, and computed the extracellular potential field created by a 1-µA stimulus for different values of tissue conductivity ranging from 0.05 to 1.65 S/m (Figure 7.A). These simulations predict that the less conductive the neural tissue, the higher the electrical potential near the stimulation electrode. This can be explained by the fact that near the stimulation electrode, the conservation of the current (*σ_tissue_*×∇*V*) imposes greater variations of the potential for lower conductivities, hence greater values of the potential. By contrast, for distances beyond about 1000 µm from the stimulation electrode, the potential field is not affected by the conductivity of the neural tissue. This result can be explained by the fact that far away from the stimulation electrode, the potential is imposed by the ground electrode, which is always surrounded by the Ringer solution.

Second, we recorded experimentally the potential field generated by a current-controlled command stimulation of 1 µA, in the presence of a whole embryonic mouse hindbrain-spinal cord preparation (Figure 7.B), and adjusted the model parameters (*g_stim_*, *g_ground_*, and *σ_tissue_*) to fit these data. The regression slope and intercept were 0.987±0.014 and 7.39±6.66 µV, respectively (R^2^ = 0.989, p<0.0001). We estimated the following optimal parameters: *g_ground_* = 799 S/m^2^, *g_stim_* = 116 S/m^2^, and *σ_tissue_* = 0.057 S/m. Adjusting *σ_tissue_* thus provided an estimation of the conductivity of the neural tissue. The value of *g_ground_* was relatively close to the one fitted in the absence of tissue (*g_ground_* = 975 S/m^2^), which was consistent with the fact that the solution in front of the ground electrode was unchanged in both cases. By contrast, the value of *g_stim_* was lower than that obtained without tissue (116 S/m^2^ vs. 338 S/m^2^).

### 5. Improvement of stimulation focality with a new electrode configuration

The second major goal of this paper was to study the stimulation focality for different electrode configurations, and to propose a new configuration that improves the focality of both the potential field and the threshold-distance curves for neurons placed in this field. For this purpose, we used the model based on Robin BCs described and validated above. The way to improve the focality of a stimulation is to constrain the current to flow back through some location close to the stimulation electrode. In this respect, multipolar electrode configurations are generally considered [13], [15], [17], [35]–[38]. Here, we considered the standard monopolar configuration, a concentric bipolar configuration, and also a new electrode configuration consisting of a ground surface (GS) surrounding the electrodes of the array (see Figure 3). We modeled the ground surface as a filled plane, only open at the location of the stimulation electrode, although it could have a different shape, such as a grid, or a network of interconnected counter-electrodes. We checked that the results would not have differed significantly with such shapes.

**Figure 8:**
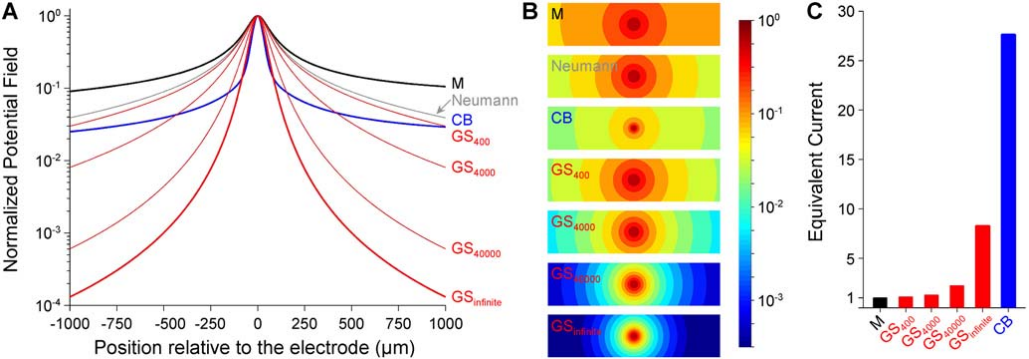
Improvement of the focality of the potential field with a ground surface configuration. The normalized potential field (*V_norm_*) is plotted along a line passing 50 µm over the electrodes (A), and mapped over the *z* = 50 µm horizontal plane (B). The offset values were: 0 mV for the monopolar configuration and the concentric bipolar configuration, and 0.027, 0.00044, 0.0000026, and 0 mV for the ground surface configuration, when using Robin boundary conditions with *g_GS_* = 400, 4000, 40000 S/m^2^, and infinite. The maps cover a distance of 1000 (respectively 250) µm on both sides of the stimulation electrode, in the *x* (respectively *y*) direction. The three electrode configurations presented in Figure 3 are considered: Monopolar (M), Concentric Bipolar (CB) and Ground Surface (GS). For this latter configuration, the surface conductance *g_GS_* was assigned four different values: 400, 4000, 40000 S/m^2^, and infinite. We also characterized the focality obtained using non homogeneous Neumann BC (Equation 13), which corresponds to a very low conductance of the ground surface. For a nominal current of 1 µA, the maximum potential field was 1.99 mV 50 µm above the planar disk electrode in the case of a monopolar configuration. Panel C shows the equivalent current required to reach the same potential amplitude with each configuration.

In a first step, we assessed the focality of the normalized potential field for these three configurations (Figure 8.A&B). As expected, we found that the concentric bipolar (CB) configuration generated more focal potential distributions than the monopolar configuration (M). However, with this configuration, the maximum amplitude of the potential was 26.7 times smaller than that obtained for the monopolar configuration for the same current. Thus, using such CB configuration, much higher currents would be required to achieve the same level of potential near the stimulation electrode (see Figure 8.C). Moreover, the CB configuration also requires doubling the number of electrodes of the array.

For these reasons, we tested the use of a ground surface laying on the substrate of the MEA around the electrodes. We found that this configuration increases the potential field focality (see GS plots and maps in Figure 8), and that the stimulation focality improved as the surface conductance *g_GS_* of the ground surface increased, the best focality being obtained in the limit case of an infinitely conductive ground/electrolyte interface (modeled with a homogeneous Dirichlet condition). Moreover, the ground surface approach leads to a reduction of the potential field amplitude with respect to monopolar stimulation by factors of only 1.10, 1.30, 2.22, and 8.30 for *g_GS_* = 400, 4000, 40000 S/m^2^ and infinite, respectively.

**Figure 9:**
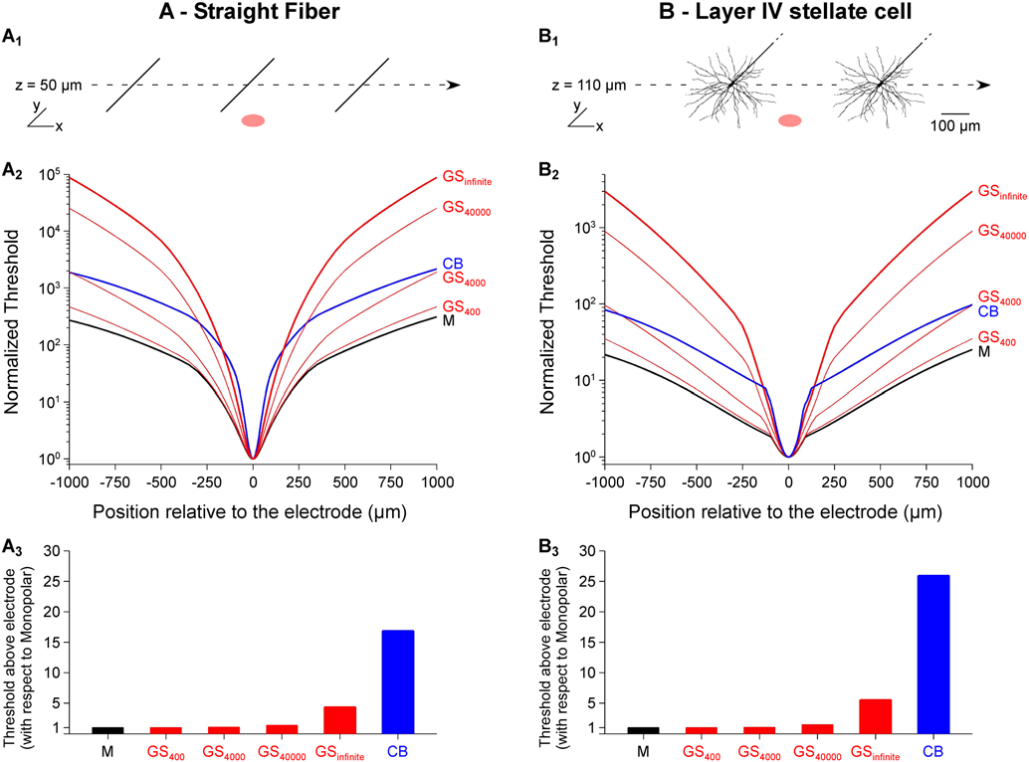
Improvement of the focality of threshold-distance curves with a ground surface configuration. Current thresholds required to activate a straight fiber (A1) or a cortical stellate cell (B1) were determined along a line passing over the electrodes, and normalized by the minimum threshold value along the line, for each configuration. A2 and B2: Normalized thresholds for all electrode configurations. A3 and B3: Thresholds above electrode for all configurations, normalized with respect to that obtained for monopolar stimulation (94.1 µA for the fiber, 436 µA for the stellate cell). This normalization allows comparing directly the factor by which stimulation current should be increased with respect to the monopolar case for the different electrode configurations. It clearly appears that the CB requires much stronger currents than the ground surface approach.

In a second step, we assessed the focality of the stimulation of each configuration in the case of a straight fiber (Figure 9.A1&A2) and a reconstructed cortical neuron (Figure 9.B1&B2). In both cases, we found that the ground surface configuration strongly improves the stimulus focality, especially for high interface conductance. Another point is that the focalization of the threshold-distance curves provided by the GS configuration is not limited to the first hundred of microns around the stimulation electrode, as it is the case for M and CB stimulations. Also and more importantly, the GS approach requires less than two times higher currents to stimulate a cell (for *g*_GS_ = 400–40000 S/m^2^) compared to the monopolar case, while the CB approach requires 17–26 times higher currents (Figure 9.A3&B3).

## Discussion

The goal of this study was both to validate a new model for the computation of the potential field created in the extracellular medium by an electrical microstimulation, and to develop a novel electrode configuration allowing focal stimulations.

In this paper, by comparing experimental recordings and modeling results, we first showed that accurate calculation of the extracellular potential created by an electrical stimulation can be achieved using a finite element model equipped with Robin BCs on stimulation and ground electrodes. In particular, we found that it is important to take into account the potential drop at the stimulation and ground electrode/medium interfaces. We verified (data not shown) that accounting for this drop at the stimulation electrode but not at the ground electrode (i.e. at which *V* = *V_metal_* = 0), did not allow an accurate computation of the potential field (regression slope of 1.28±0.005 and intercept of −171±3.36 µV). By contrast, taking into account the potential drop at both interfaces provides a good estimation of the potential field.

As shown in Figure 5, the potential field *V* in front of the stimulation and ground electrodes is actually non-uniform. As the surface conductance of the interface increases, this non-uniformity disappears and the medium voltage tends to take uniformly the metal voltage of the electrode.

When *g_ground_* increases, the medium voltage *V* becomes more uniform and close to zero in front of the ground electrode. For current-controlled monopolar stimulations, this has no influence on the potential shape recorded on distant electrodes (as in the classical monopolar case), except a change on the overall offset (Figure 6.A). However, when a ground surface surrounding the electrodes of the array is considered, *g_GS_* strongly influences the shape and focality of *V* (Figure 8).

When *g_stim_* varies, the potential field on the recording electrodes does not change as long as the stimulation current remains unchanged (Figure 6.B). However, very locally in front of the stimulation electrode surface, the potential distribution does actually vary when *g_stim_* varies (Figure 5.B). We found that the averaged value of *V* in the medium over the surface of the stimulation electrode (<*V*>*_stim_*) remains however constant as *g_stim_* varies. The evolution of the metal voltage *V_stim_* as a function of *g_stim_* can be further described analytically by integrating the Robin BC (Equation 5) over the stimulation electrode:
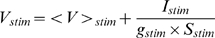
(12)Overall, these results mean that, for a given current, when *g_stim_* increases, the stimulation electrode voltage (on the metal side) *V_stim_* decreases so that the current and the average potential on the medium side, as well as the potential field away from the electrode, remain constant.

The use of Robin BCs requires knowing the values of the electrodes' surface conductance, which depends on many factors, such as for example the electrode material or the stimulation frequency. This BC (Equation 5) can actually be simplified when the surface conductance *g_stim_* is small enough so that the potential drop at the interface is high, namely *V*≪*V_metal_*. In this case, the Robin BC becomes *σ*∇*V*·**n** = *g V_metal_*−*g V*≈*g V_metal_*. Integrating this expression over the electrode surface leads to *g V_metal_* = *I*/*S*, and thus to the non-homogeneous Neumann condition:
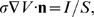
(13)where *I* is the injected current, and *S* the electrode surface.

In practice, this simplification is valid for the type of Pt stimulation microelectrodes used in the present study. We actually found (see Figure 5.B) that the metal voltage of the stimulation electrode (*V_stim_* = 754.4 mV) was much higher than the potential on the medium side (*V* ranging from 4.9 to 9.5 mV), meaning that the potential drop was nearly uniform. We then verified that the use of this simplified BC gave similar simulation results to those obtained with the Robin BC. This non-homogeneous Neumann BC would be very useful when surface conductances or electrode voltages are unknown, since it requires only the knowledge of the current and the area of the stimulation electrode. Nevertheless, it should be noted that this simplified BC is not valid for the ground electrode, over the surface of which *V* is highly non-uniform (Figure 5.A), so that, since the metal voltage on the ground electrode is zero (*V_ground_* = 0), the potential drop (and thus the current density) is also non-uniform. This may not induce large differences in the calculation of the potential when the ground electrode is located far from the region of interest. However, in the case of the ground surface configuration, which surrounds closely the stimulating electrodes, using this BC would not have allowed seeing the potent influence of the surface conductance of the ground surface on the focality of the stimulation (see gray curve in Figure 8.A and corresponding 2D map in Figure 8.B). More precisely, while the Dirichlet BC corresponds to the extreme case of an infinitely conductive electrode-medium interface (maximum focality with the ground surface, see the thick red curve in Figure 8.A), the non homogeneous Neumann BC corresponds to the opposite limit case of a highly resistive interface (minimum focality).

The results reported in Figure 6 have important practical consequences when designing stimulation protocols. While the impedance of the stimulation electrode has no effect on the potential field for current-controlled stimulations, this quantity strongly influences the potential field obtained by voltage-controlled stimulations: The higher the impedance, the weaker the stimulation (Figure 6.C). This result explains the changes in the Volume of Activated Tissue (VAT) observed by others during DBS as a function of the electrode impedance [39]. This means that the potential field is directly determined by the current injected through the stimulation electrode, and not by the metal voltage applied to this electrode. Although recent MEA studies [40], [41] and common clinical practice [42] use voltage-controlled stimulations, our results show that current-controlled stimulations should be preferentially used in order to control the amplitude of the potential field created in the extracellular medium. Indeed, in practice, microelectrode impedances typically vary across a given array by factors on the order of 1–3, and may increase with time due to a progressive degradation of the electrodes, even during a single experiment (personal observation). Using chronic voltage-controlled stimulations may thus create potential fields unstable along time and dependent on the electrode chosen for stimulation.

The electrode/medium interface has a complex frequency-dependent impedance that can be modeled with several capacitive and resistive elements in series and/or in parallel to each other [43], [44]. For the purpose of the present study, the full knowledge of the frequency dependent behavior of the electrode, as introduced in FEM by others [45], was not mandatory. By adjusting the model parameters so as to best explain the experimental potential field, we could estimate the surface conductance of both the Pt stimulation electrode and the Ag/AgCl ground electrode at a given latency at the end of the cathodic phase of the pulse stimulus. We actually checked that, for a 1000-Hz stimulation, these estimated values were compatible with electrode impedance measured experimentally (60.9 kΩ versus 65 kΩ). This approach may thus be used to estimate the surface conductance *g* for multiple sinusoidal stimulations at different frequencies or at all time points during the square pulse stimulation, in order to obtain an estimate of the complex impedance of the electrode (this was however beyond the scope of this paper, but could be integrated in future developments of the model).

We found that the model could also be used to predict the conductivity of the neural tissue laid on the microelectrode array. This is an interesting side-result of the present work, because authors usually take conductivity values from standard studies of the literature to compute the electrical potential generated in a tissue by an extracellular stimulation [39], [46]. Here, fitting the modeled potential field to the experimental one provided a direct way to estimate the conductivity of the hindbrain-spinal cord preparation we used. We found a conductivity of 0.057 S/m, which is in accordance with, although slightly smaller than, previously published conductivity values of the CNS usually ranging from 0.083 to 0.33 S/m [47]–[49].

The second goal of this work was to estimate the focality of the potential field and of threshold-distance curves for different electrode configurations. Conventional bipolar configurations with two nearby electrodes actually focalize the stimulation, but create anisotropic potential fields [13], [24]. A way to achieve isotropic spatial stimulations is to consider a concentric bipolar pair of stimulation electrodes [19], [30]. This configuration creates a more focal field than with monopolar stimulation, but requires much stronger currents to achieve comparable amplitudes of the potential field (Figure 8) and stimulation thresholds for either fibers or neurons (Figure 9). This is a limiting drawback of this approach when considering chronic microstimulations, because conventional microelectrodes allow injecting only small currents to avoid electrode or tissue damage [50]–[52]. Moreover, the CB approach requires doubling the number of electrodes of the array, bringing constraints on the MEA microfabrication and associated electronics.

For this reason, we proposed an extension of the classical monopolar configuration that does not require additional electrodes and appears to ensure a good trade-off between stimulation focality and amount of required current. This new configuration consists in replacing the distant ground electrode by a ground surface (GS) integrated on the MEA substrate and surrounding the electrodes of the array. By contrast with classical multipolar configurations, where several electrodes must be addressed together to form a single stimulation site, this configuration enables that each electrode be used independently of the others as an individual “stimulation pixel”, in the same way as pixels of a computer screen are addressed separately. Interestingly, we found that the focality of the potential field and activation thresholds achieved with this configuration was strongest for highest surface conductance of the ground surface. This can be explained intuitively by the fact that the current always searches the least “costly” route to enter back into the ground. For a low ground conductance, the cost to travel further through the extracellular space would be small compared to the effort required to enter the ground electrode, and the stimulation would not be focal. Conversely, for a high ground surface conductance, the main cost would be to flow through the extracellular space. In this case, the current would thus return through the ground electrode at a location close to the stimulation electrode, and the stimulation would be focal. In addition, it can be noted that the ground surface configuration generates a low stimulation artifact (which is actually the extracellular electrical potential field *V*). This is an interesting property of the novel configuration, since extracellular recordings are often greatly contaminated by this artifact. Here, we estimated the surface conductance of Pt and Ag/AgCl electrodes to be 338 S/m^2^ and 975 S/m^2^, respectively. With these materials, the proposed configuration already improves the focality compared to a monopolar configuration. However, better focality would be obtained with higher surface conductances (Figures 8 and 9), such as those achievable with porous materials such as black platinum [53]. It is also worth noting that using the GS approach should allow to further adjust the focality of the stimulation by varying the pulse width. Indeed, we found that the highest the GS interface conductance, the strongest the focality. Given the capacitive property of the interface, the conductance increases when the stimulus frequency increases. Consequently, it is expected that short pulses (high frequency contents) lead to more focal stimulations than longer pulses.

Finally, one practical advantage of the proposed configuration is the trade-off it offers between stimulation focality and required current. Indeed, better focality can be achieved with currents less than two times higher than with the monopolar configuration, while much stronger currents (about 17–26 times stronger) are needed with the concentric bipolar configuration. This gain in current amplitude is important to reduce electrode deterioration and to design low-consumption implantable devices for which battery life is an important practical issue.

In conclusion, a realistic model has been validated for the computation of the extracellular potential field generated by an electrical stimulation in a neural tissue, and a new electrode configuration has been proposed to achieve focal stimulations. Based on our simulation results, we encourage modelers to use Robin BCs instead of Dirichlet BCs on the conductive electrodes, and experimenters to prefer current-controlled stimulations to voltage-controlled stimulations, in order to better control the spatial extent of the stimulations. Finally, the new configuration proposed here could be advantageously used *in vitro* to study the activity-dependent dynamics and plasticity of neural networks, and could also be adapted *in vivo* for the development of neural prostheses.
